# Novel Constrained Dual Mobility Hip Prosthesis to Combat Instability in Revision Total Hip Arthroplasty whilst Preserving Normal Function

**DOI:** 10.1155/2020/6390310

**Published:** 2020-07-07

**Authors:** Michael Jiang, Anton Lambers, Rodney Richardson

**Affiliations:** Austin Health, Melbourne, Australia

## Abstract

We present the case of a male patient with sepsis and a chronic discharging sinus in a multirevised total hip replacement. Following extensive debridement, the reimplanted hip became unstable. With the patient's long-term desire to return to work and ride a bicycle with his children, the patient agreed to proceed with a novel, custom-designed, constrained dual mobility liner which allowed unrestricted movement. In 2017, the patient underwent revision surgery with this novel dual mobility constrained prosthesis. The patient was very quickly able to regain independence. After 16 years of unemployment, he managed to return to gainful employment as a cleaner rapidly regaining function as well as finally being able to ride a bike with his children for the first time.

## 1. Introduction

Instability post Total Hip Arthroplasty (THA) is a well-recognised complication of the procedure [1]. Despite the decrease in dislocation rate in recent years, with rates as low as 0.3% reported, the rising number of these procedures worldwide has resulted in an increasing burden of the condition [2]. Early instability is often effectively managed with closed reduction, bracing, and precautions along with patient education; however, a portion of these patients develop chronic instability [2–4]. Chronic instability can necessitate invasive intervention depending on the contributing factors. This can involve introducing constrained liners to compensate for a lack of supporting anatomy in patients with an acceptable acetabular fixation and orientation [2, 3, 5, 6]. In cases due to malpositioned implants, replacement of all components may be required [7]. The difficulty in management lies in recurrence of instability post revision which can lead to a cascade of further surgery [8]. Incidence of instability and recurrent dislocation has been reported to be as high as 25% in revision THA [2, 9, 10]. As a result, research and development continues in search of suitable prostheses to combat this complication.

Deciding on the correct treatment for patients experiencing instability with appropriately positioned implants and no indication of component failure proves difficult [11]. Operative options include fitting of a constrained liner, revision THA with a larger femoral head in a standard prosthesis, or a dual mobility interface [9, 11]. Each is aimed at increasing the physical parameters required to dislocate, with common focuses being jump distance and reducing levering due to impingement [12–14], achieved by increasing the head-neck ratio. Consensus is yet to be reached regarding a gold standard management option, with each providing a distinct set of risks and benefits. The decision must be made on a case-by-case basis [11].

Hip prostheses with a larger femoral head increase the jump distance required for dislocation. Due to this mechanical advantage, prostheses with a femoral head size above 36 mm dislocate less frequently [1, 2, 7, 14]. Increasing the head-neck ratio also increases the range of motion (ROM) before impingement [5, 7]. However, these prostheses carry an increased risk of trunionosis and liner wear which is associated with osteolysis and liner fracture [8, 15]. Despite the introduction of highly cross-linked polyethylene (HXLPE), these failures are attributed to larger femoral heads which result in increased volumetric wear and thinner liners which are at risk of fracture [4, 12].

Constrained liners capture the femoral component, increasing the lever out force required to dislocate [16]. Constrained liners are designed to compensate for the lack of native structures that confer stability [16, 17]. Constrained liners typically capture the head in the polyethylene by having the polyethylene coverage extend beyond the equator of the head. The degree the polyethylene extends beyond the equator increases the strength of the construct but limits the range of motion of the hip joint. By capturing the femoral head at its terminal range of motion, the liner increases the amount of force required to lever out and dislocate. Although being the standard option in these patients, by design, constrained components significantly restrict the range of motion as the liner to neck distance is decreased [3, 7, 12]. Failure mechanisms usually relate to disruption of the containment mechanism. Wear and ultimate failure of the liner due to repeated impingement of the femoral stem on the liner is a common cause of failure [2, 18]. If the lever out force exceeds the containment force of the constraint, dislocation also occurs which cannot be enlocated in a closed fashion. Furthermore, abutment of the neck on the liner transfers these forces to the acetabular shell which can also lead to loosening of the cup [11]. Newer iterations feature a cutaway design to allow a greater range of motion [19] although use is technically demanding as minor error in positioning of the implant leads to increased impingement [16].

Conventional dual mobility (DM) implants utilise a tripolar design in which the femoral head is situated within a constrained, head-shaped polyethylene liner which in turn is seated in an unconstrained acetabular socket [9]. This design confers benefits of the stability of a large femoral head but the wear characteristics of a small head. The femoral head and liner construct enlarge the overall size of the femoral head, increasing the jump distance and force required to dislocate [11, 20]. The constrained liner component captures the femoral head whist maintaining the range of motion through the two mobile interfaces [11]. DM implants were originally designed for use in primary THA in high-risk populations, including obese patients, tumoural disease, neurological disease, and femoral neck fracture, and in revision THA [20, 21]. Dislocation rates are demonstrably lower with DM application in these populations [10]. Meta-analysis has also shown dual mobility implants to be superior to other options for revision and dislocation [10, 15]. Whilst there was a theoretical risk of increased wear due to the second interface [21, 22], studies have shown that the double-interface construct creates less wear overall, and as such, rates of aseptic loosening have decreased with newer designs. Due to the unconstrained nature of these implants the liner-socket articulation is still able to dislocate. Despite good published data on the rates of dislocation of dual mobility design, 3% of primary THR performed in the USA still suffer from instability [6].

**Figure 1:**
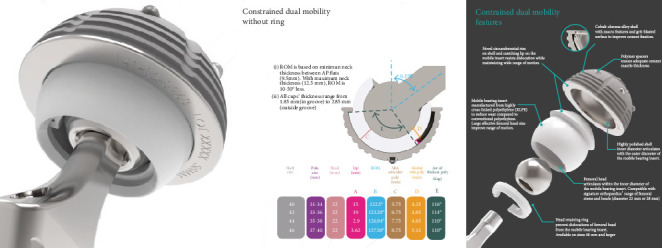
The Inovaris implant.

**Figure 2:**
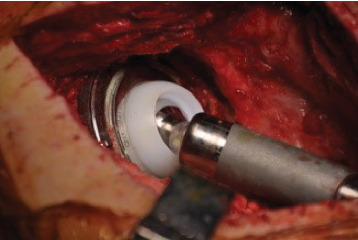
Novel prosthesis in vivo.

**Figure 3:**
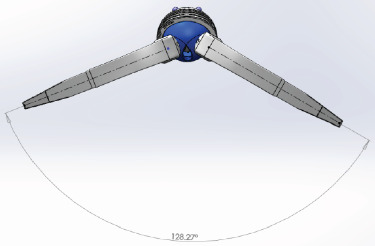
Dual mobility hip system ROM from FDA standard testing.

The novel Inovaris prosthesis (Figure 1) presented in this study features a cemented tripolar prosthesis that combines dual mobility and constraint. The design consists of a cobalt-chrome acetabular component with an inner locking rim, a highly cross-linked polyethylene liner, and a femoral head. The liner design features a containment mechanism with a unidirectional fit into a rim on the acetabular shell. This system provides unrestricted mobility through all ranges of motion with secure constraint at the terminal range of motion within the implant itself. The femoral head snaps into the polyethylene liner, secured by a locking ring. The range of motion achieved before component impingement was a 128-degree arc through the two interfaces (Figures 2 and 3). The acetabular component is able to be cemented directly into an existing, acceptably positioned cup, thereby preventing the need to revise the acetabular component altogether as seen with larger head implants and traditional DM components [1, 2].

**Figure 4:**
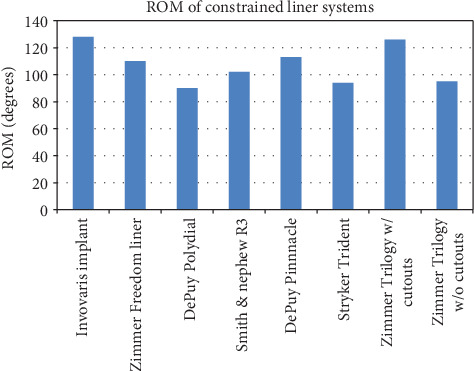
ROM of constrained liner systems currently on the market compared to the Inovaris implant.

**Figure 5:**
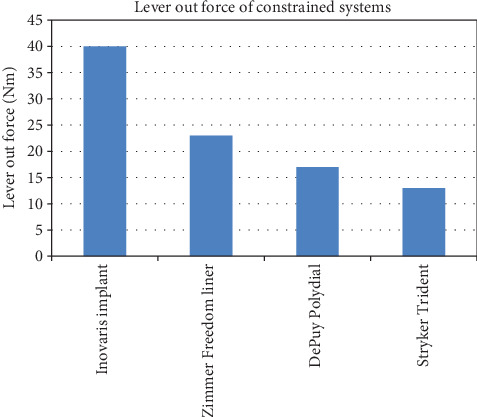
Lever out force of constrained systems.

Figures 4 and 5 compares the range of motion and lever out forces of the novel implant compared with others currently on the market as adapted from prosthetic implant product information guides based on their designs [23–26].

Post THA, there is a correlation between the hip range of motion and the hip function [27]. Patients with hip flexion above 115 degrees were found to have drastically improved function compared to those with 90 degrees or less. Many activities of daily living (ADLs)require hip flexion of up to 100 degrees with sporting activities such as cycling requiring significantly greater ROM. The restrictions imposed by current constrained systems may have detrimental effects on patient function [28]. Current systems, especially those involving cutaways, require perfect positioning of components to provide the functional range of motion for ADLs. However, any minor malpositioning will result in impingement and restricted function. This novel prosthesis was designed to address these issues. The range of motion has been increased to allow for a normal hip range of motion, even allowing for mild component malpositioning. By increasing the range of motion, there is no rim or component impingement with normal activities, thereby decreasing potential wear of constrained components. At the point of impingement at extremes of motion, the lever out strength of the construct is greater than the industry standard. The 128-degree arc is also an improvement on current constrained systems on the market along with the lever out strength.

## 2. Case Report

**Figure 6:**
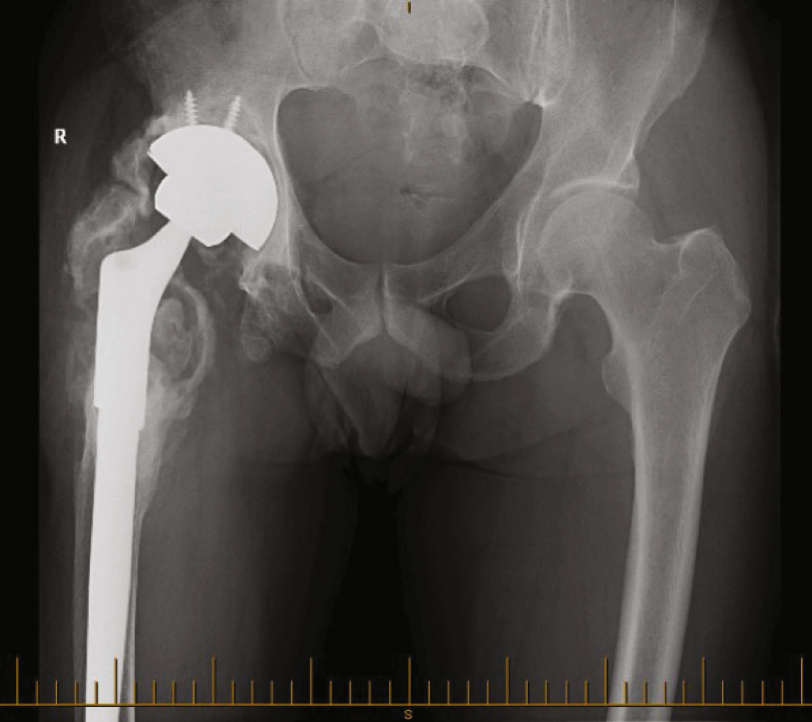
Pelvic X-ray of the patient before the 2016 operation.

A 54-year-old gentleman first presented to our practice in 2016 with a chronic deep prosthetic infection and discharging sinus. The original surgery was an open reduction and internal fixation (ORIF) performed in 1995 following a traumatic fracture dislocation of his right hip sustained in a motor vehicle accident. He subsequently developed posttraumatic osteoarthritis, and in 1996, he underwent conversion to a THA. The first THA was complicated by a dislocation three days postoperatively which required and open reduction. Due to increasing pain, a revision THA was performed in 2003. This surgery was complicated by a deep periprosthetic infection which failed to resolve after multiple washouts and prolonged antibiotics. In 2011, the patient underwent the first of a two-stage revision for infection, with reimplantation in 2012. Despite a total of 26 surgical procedures being performed, the patient was left with a chronically infected THR. In 2016, he presented with pain and a chronically discharging sinus (Figure 6).

**Figure 7:**
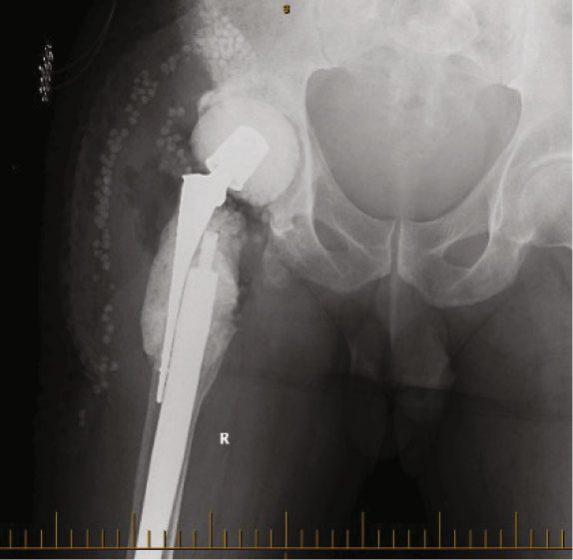
Pelvic X-ray of the patient post first-stage revision with cement spacer and retained stem 2016.

**Figure 8:**
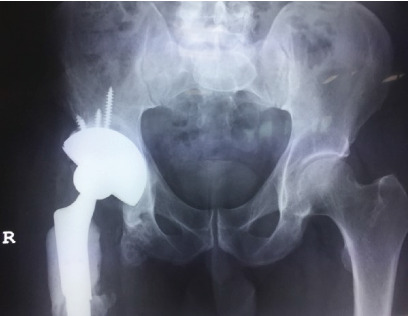
Pelvic X-ray post reimplantation procedure in 2017.

In 2016, the patient underwent a modified 2-stage revision, with retention of a solidly fixed long uncemented stem (Figures 7 and 8).

In 2017, the patient's hip function had improved. Pain had settled, and there was no discharging sinus. However, the patient reported difficulty mobilising and subjectively felt unstable.

**Figure 9:**
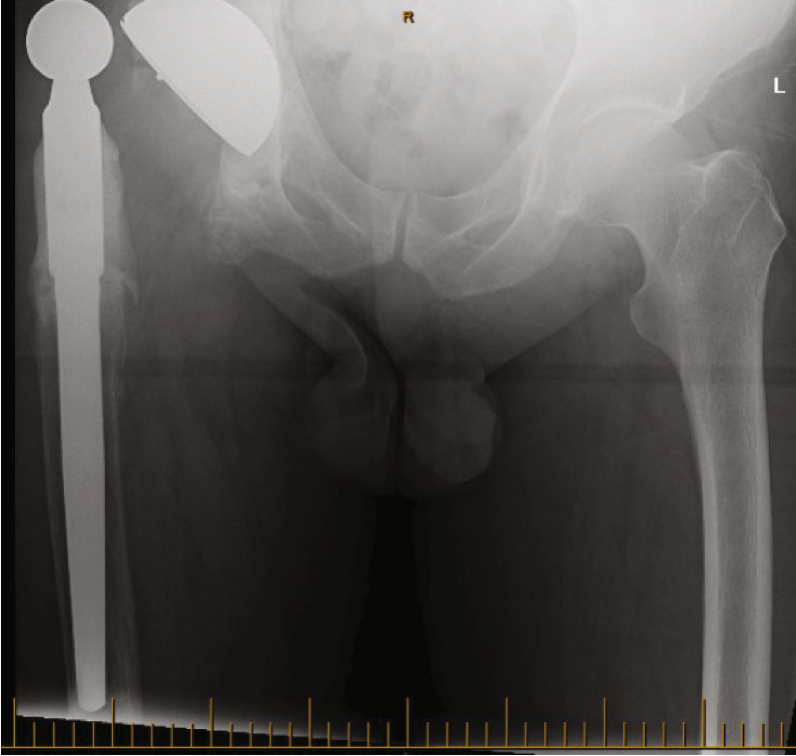
Pelvic X-ray 2017 showing dislocation of the revision prosthesis.

The patient had been extensively counselled on the high risk of instability and dislocation following the previous procedures due to the structural disruption caused from debriding infected bone. Following reimplantation of the THR in late 2016, the patient continued to report instability in the hip and two episodes of dislocation (Figure 9) which prompted the decision to opt for revision with the Inovaris prosthesis.

## 3. Operation

**Figure 10:**
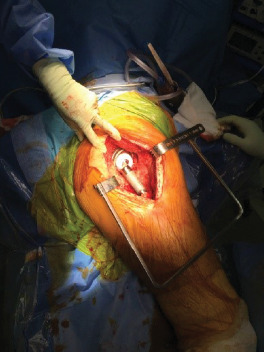
Intraoperative view of the implanted Inovaris prosthesis.

**Figure 11:**
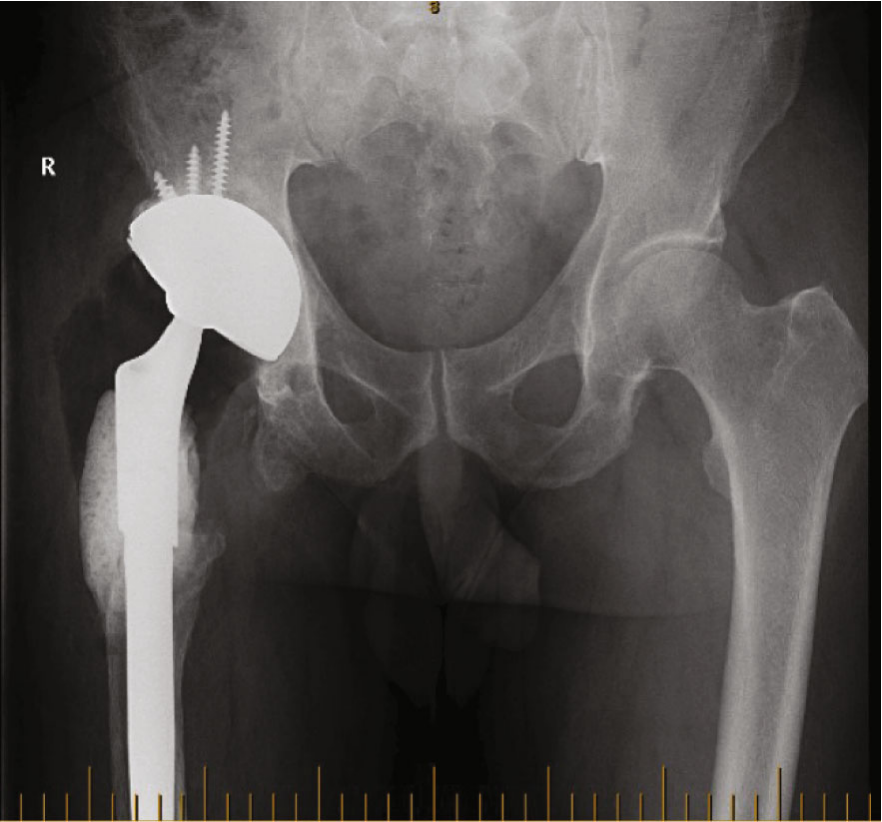
Pelvic X-ray 2017 post revision with the Inovaris prosthesis.

The revision THA was performed under general anaesthesia using a direct lateral approach (Figure 10). Due to the absence of proximal femoral bone and gluteal deficiency, acetabular exposure was achieved without difficulty. The previous polyethylene liner was removed, and pulse lavage with Betadine was used to irrigate the entire wound space. The acetabular cup was then dried and prepped for cementing of the revision acetabular device into the solidly fixed 76 mm multihole Tritanium shell. A mix of Palacos antibiotic cement with the addition 1 g of vancomycin was used. The previous femoral head, 36 mm/0, was replaced with a 28 mm/0 head. The dual mobility polyethylene liner was secured onto the femoral head and the hip reduced. Antibiotic cement was placed around the proximal stem to help minimise dead space and to deliver high-dose antibiotic. Postoperatively (Figure 11), the patient was allowed to mobilise as soon as tolerated allowing full weight bearing.

## 4. Postoperative Outcome

**Figure 12:**
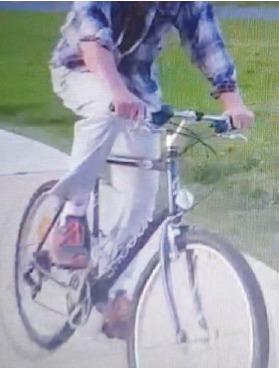
Patient riding a bicycle post revision with Inovaris implant.

**Figure 13:**
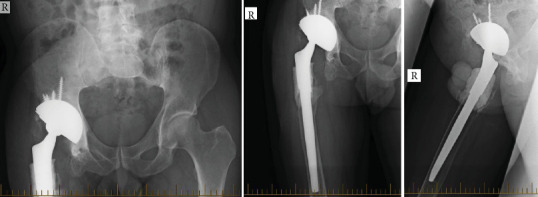
Follow-up X-rays 2020.

Following the operation, the patient reported a significant increase in level of function from his preoperative baseline. The patient was walking independently day one post-op. He reported immediately feeling more secure and was able to sit on low chairs and toilet seats in a normal posture. In the early postoperative period, he was able to run short distances and started riding a bike again (Figure 12). He could now comfortably sit in a low chair and started using a ride on mover and tractor activities which were severely limited prior to the operation. His ability to perform ADLs was also improved with the patient able to dress and bathe himself without any aid. On examination, a significant Trendelenburg gait remains as expected in the absence of abductor musculature. The patient demonstrates 50 degrees of active flexion increasing to 70 degrees passively and over 20 degrees of abduction, adduction, and external rotation. There was no significant deformity or leg length discrepancy noted. The X-rays from the most recent outpatient review in 2020 are shown in Figure 13. Two patient-reported outcome measures (PROMs) were undertaken 18 months post revision, with the Harris Hip Score (HHS) reported at 78.9 and the 36-item Short Form Health Survey (SF-36) showing significant physical impairment with 40% physical function, 50% limitation due to physical health, 45% energy/fatigue, and 55% pain scores, with overall health at 70%. He remains on antibiotic suppressive therapy but remains well.

## 5. Discussion

Chronic instability following THA can be broadly classified into those caused by malpositioned components, insufficient abductor complex, impingement, or polyethylene wear [29]. In this case, the patient's instability was on a background of deficient abductor complex, chronic infection, and appropriately positioned implants. We present the novel dual mobility device as a range preserving constrained option for treatment of instability.

The recommended treatment in this case was to use a conventional constrained liner [3]. However, this carried restrictions on function incompatible with the patient's wishes. Functionally, the patient wanted to be able to be independent with activities of daily living and be able to participate in activities such as riding a bicycle. Restrictions on the range of motion imposed by current implants would not allow this level of use of the limb. Revision with a larger femoral head or conventional dual mobility device also provided barriers. Given that the previous two revisions with conventional THA proved unsuccessful, it was unlikely that a further revision would improve symptoms. It was considered unlikely that a conventional dual mobility implant would provide the stability required in the setting of proximal bone loss. The Inovaris implant was chosen to allow unrestricted movement and strength of containment. Implantation of the construct directly into the existing, well-positioned cup avoided the extra steps necessary for alternative devices. The range of motion afforded by the device allowed independent function.

Functionally, the device has performed as expected. Instability is no longer a concern, a significant finding given the previous history and extensive disruption of the anatomy surrounding the hip joint. Independence in ADLs has been reported, along with running and riding a bicycle within the 12 months following revision. The patient has also reported the ability to sit for prolonged periods of time on a tractor, which he had not been able to do previously. The patient has also returned to gainful employment. Given the previous results following revision, we postulate that these results would not have been possible with other devices currently available.

Whilst this case report demonstrates successful use of this implant in treating instability post revision THA, further research is required to quantify the complication rate and definitive change in the range of motion and PROMs pre- and postoperatively.

In summary, we present the first use of a novel dual mobility hip prosthesis in the management of recurrent instability in the setting of chronic infection. At 1-year follow-up, instability is no longer of major concern to the patient and improvements in quality of life and function have been reported. We present this prosthesis as a viable treatment option in cases of instability following revision THA, with advantages of construct strength, range of motion, and decreased liner wear compared to current options on the market.
